# Metabolic and Clinical Outcomes in Type 1 Diabetes in the COVID-19 Pre- and Post-Vaccination Periods in Spain: The COVID-SED1 Study

**DOI:** 10.3390/jcm13071922

**Published:** 2024-03-26

**Authors:** Fernando Gómez-Peralta, Edelmiro Menéndez, Santiago Conde, Pablo Abellán-Galiana, Miguel Brito, Marina Beléndez, Antonio Pérez

**Affiliations:** 1 Endocrinology and Nutrition Unit, Hospital General de Segovia, 40002 Segovia, Spain; 2 Endocrinology and Nutrition Service, Hospital Universitario Central Asturias, 33011 Oviedo, Spain; edelangot@gmail.com; 3 Centro de Salud de Barbastro, 22300 Huesca, Spain; seisdoble26@hotmail.com; 4 Department of Endocrinology and Nutrition, Hospital General Universitari de Castelló, 12004 Castellón, Spain; abellan_pab@gva.es; 5 Department of Medicine and Surgery, Universidad Cardenal Herrera-CEU, 12006 Castellón de la Plana, Spain; 6 Endocrinology and Nutrition Service, Hospital Puerta de Hierro, 28222 Madrid, Spain; 7 Departamento de Comunicación y Psicología Social, Universidad de Alicante, 03690 Alicante, Spain; marina.belendez@ua.es; 8 Servicio de Endocrinología y Nutrición, Hospital de la Santa Creu i Sant Pau, 08025 Barcelona, Spain; 9 Institut de Recerca de Sant Pau (IIB Sant Pau), Universitat Autònoma de Barcelona (UAB), CIBER de Diabetes y Enfermedades Metabólicas Asociadas (CIBERDEM), 08193 Barcelona, Spain; 10 SED1 Study Investigators, Sociedad Española de Diabetes—SED, 28002 Madrid, Spain

**Keywords:** type 1 diabetes, COVID-19, vaccination, glycemic control, continuous glucose monitoring, diabetes complications

## Abstract

**Aims:** To evaluate the metabolic and clinical outcomes in the Spanish type 1 diabetes mellitus (T1D) population before and after COVID-19 vaccination. **Methods:** A retrospective observational study was carried out in Spanish public hospitals previously enrolled in the SED1 study. Adults and children with T1D were included and their clinical electronic records were reviewed. Clinical, laboratory, and glucometric parameters from continuous glucose monitoring (CGM) data corresponding to the periods before and after administering the first COVID-19 vaccination were analyzed. **Results:** A total of 26 centers and 228 patients participated in this new phase of the SED1 study and 187 were finally evaluable (mean age 37.5 ± 15.6 years, 56.7% women). Overall, 94.6% of the sample was vaccinated, and this percentage increased with higher levels of education (*p*-value = 0.027). In the pre- and post-vaccination periods, respectively, the number of patients with acute hyperglycemic decompensation was 6/161 (3.7%) and 7/161 (4.3%) (*p* = 1) and with acute hypoglycemic decompensation was 6/161 (3.7%) and 6/161 (3.7%) (*p* = 1). The HbA1c level was lower in the post-vaccination period(mean ± SD, mg/dL): pre-vaccination 7.4 ± 0.9; post-vaccination 7.2 ± 1.0, (−0.19; *p*-value = 0.0006). A total of 31.9% of patients (95% CI: 24.7–39.7) in the pre-vaccination period and 45.0% (IC95%: 37.1–53.1) in the post-vaccine period had HbA1c < 7% (*p*-value < 0.001). Glucometrics from CGM data also showed numerical improvements post-vaccination. **Conclusions:** The COVID-19 vaccination was highly accepted in the Spanish T1D population, with hesitancy about the COVID-19 vaccine being higher in those with lower educational levels. A mildly better glycemic control was observed in the post-vaccination period.

## 1. Introduction

Type 1 diabetes mellitus (T1D) is an autoimmune disease against pancreatic beta-cells, reducing insulin production [1]. According to recent studies, the prevalence and incidence of T1D are increasing worldwide [2]. A recently published meta-analysis reported that T1D prevalence is 9.5 per 10,000 people [3]. 

Coronavirus type 2, causing Severe Acute Respiratory Syndrome (SARS-CoV-2) and known as coronavirus disease 2019 (COVID-19), has greatly influenced the T1D population. Diabetes is a prevalent comorbid condition in people affected by COVID-19, and it is associated with a poor prognosis [4]. COVID-19 infection obviously can affect glycemic control [5] and the lockdown after the COVID-19 outbreak affected the daily lives of the T1D population [6,7,8]. 

However, scarce data are available about the impact of COVID-19 vaccination on glycemic control and clinical endpoints in this population. A very recent study retrospectively reviewed data on vaccine safety in 72 T1D subjects and showed a similar risk of injection site pain, minor and major vaccine adverse events, as well as associated hospitalizations compared to healthy controls [9]. However, some articles reported a series of cases of acute hyperglycemia after receiving COVID-19 vaccines [10,11].

Moreover, during the COVID-19 post-vaccination period, many other factors could have indirectly influenced the overall health outcomes of the T1D population. The progressive normalization of daily life includes diet and exercise, work, or commuting changes. Significantly, the emotional and psychosocial burden of the COVID-19 pandemic affected the T1D population. Some studies showed an overall reduction in well-being during the COVID-19 pandemic, which is associated with poorer metabolic control and higher use of electronic media [12]. However, the post-vaccination period could positively affect psychosocial well-being by reducing diabetes distress, ultimately improving the clinical outcomes and metabolic control in this population.

Continuous glucose monitoring (CGM) devices allow for 24 h real-time measurement of interstitial glucose levels. The large amount of data generated by CGM can be analyzed and evaluated using a set of standardized parameters, collectively named glucometrics. CGM glucometrics proved highly useful during the COVID-19 pandemic, as shown in numerous studies that analyzed data during this period [13,14,15]. A few published studies with different approaches addressed the COVID-19 vaccination impact on CGM data with disparate conclusions [16,17,18,19].

This study aimed to evaluate clinical events, including hyper- and hypoglycemic emergencies and laboratory and CGM glucometric changes, between the COVID-19 pre- and post-vaccination periods in adults and children with T1D in a multicentric and representative sample of the Spanish T1D population.

## 2. Materials and Methods

### 2.1. Study Design

The already-published SED1 study was a multicentric, cross-sectional, observational study that included a representative sample of the T1D Spanish population treated at the endocrinology specialist consultations of 75 public hospitals in Spain [20]. Patients had to meet the following criteria: diagnosed with T1D, with a medical record at the site, with at least two HbA1c values available at the study visit, and provided written informed consent. Patients with a diagnosis of type 2 diabetes mellitus (T2D) and those with a history of pancreas and/or islet cell transplantation were excluded. From this cross-sectional study (baseline visit during the first semester of 2018), a historical cohort was constructed. During the second semester of 2022, observational follow-up data were obtained directly from the patients or their parents or from medical records pertaining to two periods pre- and post-COVID 19 pandemic and uploaded to an online electronic form. 

This study was conducted in accordance with the principles stated in the Declaration of Helsinki. Approval for the study was granted by the Spanish Agency of Medicines and Medical Devices (AEMPS) and the ethics committee of the Hospital General de Segovia (Spain) (Comité de Ética de la Investigación con medicamentos (CEIm) del Área de Salud de Segovia), protocol code SED-1(SED-INS-2017-01), Review Board/Ethics Committee code: 17-024, 29 September 2021. All participants provided written informed consent before enrolling in the study.

### 2.2. Endpoints

The primary endpoint was to describe the change in metabolic control (laboratory hemoglobin A1c—HbA1c—and time in glucose range 70–180 mg/dL—TIR) after COVID-19 vaccination in T1D patients.

Other secondary endpoints included the change in overall glycemic control and variability (glucometrics and CGM-derived variables), complications associated with T1D (hyper- and hypoglycemic episodes), and COVID-19-related events before and after COVID-19 vaccination.

### 2.3. Evaluated Variables

The evaluated variable used in this study include anthropometric variables (weight, height, body mass index (BMI), systolic blood pressure (SBP), diastolic blood pressure (DBP), and heart rate), comorbidity and complications associated with T1D (acute hyperglycemic and hypoglycemic decompensations and hospitalizations related to T1D), laboratory results (fasting plasma glucose, glycated hemoglobin A1c (HbA1c), creatinine, total cholesterol, LDL and HDL, triglycerides, liver transaminases, autoimmunity markers, and thyroid-stimulating hormone (TSH)), treatment with basal and rapid insulin and concomitant treatment, mortality and cause of mortality, COVID-19 -related variables (COVID-19 infection, hospital admission for COVID-19, days of hospitalization for COVID-19, ICU admission and number of days, and need for mechanical ventilation), vaccination against COVID-19 (date and type of vaccine administered), and glucometrics and CGM-derived variables obtained from subjects using the Freestyle Libre2 CGM sensor (daily scans average, glucose average, coefficient of glucose variation (CV), standard deviation of glucose levels (SD), % of time with glucose in range 70–180 mg/dL (TIR), % of time with glucose below 70 mg/dL (TBR70), % of time with glucose below 54 mg/dL (TBR54), events/day and duration average of hypoglycemic events with lower than 70 mg/dL, % of time with glucose above 180 mg/dL (TAR180), % of time with glucose above 250 mg/dL (TAR250), glucose management indicator (GMI)).

### 2.4. Statistical Analysis

Continuous variables include the number of patients with valid observations and mean and standard deviation (SD), or median and first and third quartile (Q1–Q3) where applicable. Categorical variables are described by number and proportion.

Differences between the periods (before and after vaccination) were examined using the paired *t*-test for continuous variables and the McNemar’s test or exact binomial test for qualitative variables. 

The chi-squared test or Fisher’s exact test was used to compare qualitative variables between patients with or without COVID-19 infection. The proportion of adequate diabetes control and 95% confidence intervals (CI) were computed. 

A *p*-value of <0.05 was considered statistically significant. Statistical analyses were generated using Stata version 12 (Stata Corporation, College Station, TX, USA).

## 3. Results

### 3.1. Population

A total of 26 centers and 228 patients accepted to participate in this new phase of the previous SED1 study [20] and 187 were finally evaluable.

Sociodemographic and clinical characteristics are described in Table 1. The mean age was 37.5 ± 15.6 years, 56.7% were women, and 97.9% were Caucasian. Additionally, 70.5% were non-smokers, and 62.0% did not consume alcohol. The mean time since diagnosis of T1D was 17.8 ± 12.7 years. Regarding family history, 35.4% had a family history of T2DM, 21.3% of T1D, and 17.7% of hypothyroidism. Likewise, 72.3% considered adherence to the diet to be good/very good. A total of 47.6% of the population with follow-up had some comorbidity and/or complications associated with T1D, with retinopathy being the most frequent (18.7%), followed by dyslipidemia (15.5%) and hypothyroidism (12.8%). Among the patients, 82.6% had episodes of hypoglycemia in the month prior to the inclusion visit. Regarding glycemic monitoring, 97.8% of the patients performed daily blood glucose self-monitoring (BGSM), with an average frequency of 4.4 ± 1.8 controls per day, and 30.6% of the patients followed used CGM. Regarding insulin treatment, the most frequent method of insulin administration in the population with follow-up was bolus-basal (74.3%), followed by continuous infusion pump (24.1%) and premixes (1.6%).

The sociodemographic and clinical characteristics of the population included in this second phase of the SED1 study are not different from the original sample [20], nor from those without follow-up (Supplementary Table S1).

### 3.2. COVID-19 Vaccination

A total of 94.6% of patients have been vaccinated against SARS-CoV-2. Of the total number of vaccinated patients, about 18% had a reaction to the first dose of the vaccine, and 6.7% reported that the first dose had an impact on glycemic control. The second dose of the vaccine was administered to 94.2% of vaccinated patients. Comirnaty (Pfizer-BioNTech, New York, NY, USA) was the most commonly administered vaccine, both in the first (75.4%) and the second (74.3%) doses, followed by Spikevax (Moderna, Cambridge, MA, USA) (13.6% and 17.4%, respectively). Among patients who received the second dose, 24.6% had side effects after receiving the vaccine and 10.7% had an impact on glycemic control. Overall, 73.2% of vaccinated patients received the booster dose. Of those, 55.8% received Spikevax (Moderna) and 44.1% Comirnaty (Pfizer-BioNTech). In 11.2% of cases, patients that perceived the booster shot had an impact on glycemic control.

The percentage of vaccinated people is higher among those with a higher level of education (*p*-value = 0.027). The percentage of vaccination according to patient education is 91.4% in individuals with primary education, 94.9% in those with secondary education, and 98.3% in people with university studies. 

### 3.3. COVID-19-Related Events

A total of 19.4% (95% CI: 13.5–26.4) were infected with COVID-19 before vaccination and 42.5% (95% CI: 34.7–50.5) after being vaccinated (*p*-value < 0.001). Among the patients who contracted the disease, two (6.45%) pre-vaccination and one (1.47%) post-vaccination were hospitalized for COVID-19. Of the patients hospitalized for COVID-19, no pre-vaccination and the one post-vaccination were admitted to the ICU and required mechanical ventilation. One death (0.53%) occurred in the pre-vaccination period and one (0.55%) in the post-vaccination period.

### 3.4. Comorbidity and Complications Associated with T1D

Differences in acute decompensation cumulative incidence among those with or without COVID-19 infection did not reach statistical significance: acute hyperglycemic decompensation, 3/71 (4.2%) and 7/85 (8.2%), respectively (*p* = 0.348); acute hypoglycemic decompensation, 3/71 (4.23%) and 6/85 (7.1%), respectively (*p* = 0.511). Regarding the pre- and post-vaccination periods, the acute decompensation cumulative incidence was as follows: acute hyperglycemic decompensation, 6/161 (3.7%) and 7/161 (4.3%) (*p* = 1), respectively; acute hypoglycemic decompensation, 6/161 (3.7%) and 6/161 (3.7%) (*p* = 1), respectively.

There is no change in cases of acute decompensation, both hyperglycemic and hypoglycemic, between the pre-vaccine period and the post-vaccine period (Table 2).

Regarding the vaccine type and acute decompensation frequency, Table 3 describes the relative frequency of the clinical events with two main vaccines used.

### 3.5. HbA1c before and after Vaccination

The HbA1c level was reduced after vaccination (mean ± SD, mg/dL): pre-vaccination 7.42 ± 0.96%; post-vaccination 7.23 ± 1.03%, (−0.19; *p*-value = 0.0006). There was a statistically significant difference in the proportion of patients who have adequate control of T1D (HbA1c < 7%) before and after vaccination: 31.9% of patients (95% CI: 24.7–39.7) in the pre-vaccination period and 45.0% (IC95%: 37.1–53.1) in the post-vaccine period (*p*-value < 0.001).

### 3.6. Glucometrics and CGM-Derived Variables before and after Vaccination

No statistically significant differences were observed in the comparison of glucometrics and variables derived from CGM at each of the evaluation moments (Table 4). A numerical improvement is observed in many CGM-derived variables in the post-vaccine period, without reaching statistical significance. It is necessary to bear in mind that the same number of patients is not available for all variables due to lack of information.

## 4. Discussion

Patients with T1D are at a high risk of poorer outcomes during COVID-19 infection [4]. Infection itself can cause hyperglycemia, including acute hyperglycemic emergencies [21,22]. Interestingly, several studies have shown that glycemic control in the overall T1D population improved during the lockdown [8,13]. One possible reason for this improvement could be a positive impact of COVID-19 vaccination. However, previous studies using CGM data showed an immediate impairment in glycemic control after the first or booster vaccination [16,17,18,23]. An observational, unicentric, retrospective study reported an absence of unexpected patterns of adverse events, and difference, in glucose in the subgroup of patients sharing CGM data after receiving the mRNA-1273 (Moderna) vaccine [16]. Another retrospective analysis of 96 adults with T1D examined the CGM data in the periods immediately before and after their first COVID-19 vaccination [17]. A total of 30% of individuals experienced a decrease in time within range of over 10%. Only a slight deterioration in immediate glycemic control was obtained in another retrospective study using CGM before and after the first and second dose of the COVID-19 vaccine in Arabic people with T1D [18]. Moreover, a prospective pilot study of adults with T1D evaluated changes in CGM glucometrics after a COVID-19 booster vaccine, suggesting transient mild glycemic elevations after it [19]. 

However, many factors apart from the direct effect of vaccines could affect the evolution of the clinical and metabolic situation of the T1D population throughout the post-vaccination period. Nutritional and physical activity patterns changed after progressive daily life normalization after lockdown. Additionally, the psychological burden caused by the COVID-19 pandemic increased the diabetes distress in the T1D population. One study among adults with type 1 diabetes in the US in 2021 showed that 41.2% experienced moderate diabetes distress and 19.1% experienced high diabetes distress [24]. Another German study observed an overall well-being reduction during the COVID-19 pandemic, associated with poorer metabolic control and higher use of electronic media [12]. However, psychological relief after vaccination could be expected. Our results point to a possible beneficial influence of COVID-19 vaccination in the meantime in the overall T1D population. A possible explanation for the discrepancy with the previous studies looking at the immediate effect of COVID-19 vaccines on glycemic control could be that the CGM and laboratory data were analyzed some time after vaccination. An immediate deterioration in glycemic control in the first days after vaccination can be followed by an overall metabolic improvement due to the reduction in the clinical impact of subsequent COVID-19 infections. Additionally, it is not possible to measure all the factors influencing clinical and glycemic outcomes in this population, and it limits the association conclusions in our study. It cannot be ruled out that the differences in the degree of control are due to changes in several variables such as diet or physical exercise since there is no information available to adjust for these factors. 

Our results showed a mild HbA1c level reduction reaching statistical significance. However, the CGM-derived glucometrics, particularly the GMI, did not reach statistical significance between both periods. The first issue to point out is that laboratory HBA1c and GMI variables are not always and completely superimposable, even in the same individual [25]. Additionally, the sample with laboratory data and the one with CGM data are not the same due to missing CGM data from some subjects. A comparison of the mean values of GMI was evaluated for 106 subjects, the number of individuals who had information on this index in both periods (pre-vaccination and post-vaccination). However, the differences in mean HbA1c values between the periods were evaluated considering 160 cases (the number who had HbA1c values collected in both periods). Finally, the limited size of the sample probably hinders the attainment of statistical significance for this association in the CGM glucometrics, despite previous results published using CGM to study the effects of the COVID-19 lockdown [13]. 

The high acceptance of COVID-19 vaccination within the Spanish T1D population is noteworthy. The COVID-19 vaccination was widely embraced among the general Spanish population, with an acceptance rate of 84.5% in May 2021, as reported in a similar age group survey [26]. The nearly universal public health system was key to the great success of the campaign in the general population and, probably, also in the T1D population. Our acceptance data of 94.6% are even better than that of the general population and higher than that observed in other nearby T1D populations. For instance, hesitancy about the COVID-19 vaccine was reported at 13% in a survey of people with T1D in Italy [27].

The limitations of our study include the retrospective design and the limited sample size. The difficult circumstances presented during the COVID-19 pandemic, especially for healthcare providers, were a barrier to finally reaching and obtaining all the expected professionals and data. However, the effort to collect data describing the underexplored post-vaccination period outcomes in the T1D population, amidst the exceptionally unusual social situation of the pandemic, should be considered. Despite the fact that the finally evaluable sample was reduced from that originally described in the SED1 study, the sociodemographic and clinical characteristics of the population included in this second phase are not different from the original sample [20], nor from those without follow-up. The availability of CGM data is another study strength. 

## 5. Conclusions

In conclusion, the first COVID-19 vaccine was widely accepted among the Spanish T1D population. The COVID-19 vaccine acceptance rate was higher in patients with higher educational levels. It appears to have produced a mild improvement in metabolic control during the post-COVID-19 vaccination period in the Spanish T1D people.

## Figures and Tables

**Table 1:** Baseline sociodemographic and clinical characteristics of the study population.

	n	%
Number of patients	187	
Age (years)		
mean ± SD		37.5 ± 15.6
Age Groups (Years)		
0–13	17	9.1
14–17	7	3.7
18–25	21	11.2
26–49	102	54.5
>49	40	21.4
Gender, women	106	56.7
Level of education	179	
No studies	2	1.1
Primary education	38	21.2
Secondary education	66	36.9
University studies or similar	67	37.4
Student	6	3.4
Weight (kg)		
mean ± SD		67.6 ± 16.8
Height (cm)	186	
mean ± SD		164.6 ± 13.1
BMI (kg/m^2^)	186	
mean ± SD		24.6 ± 4.4
BMI grades (kg/m^2^)	186	
<18.5	15	8.1
18.5–24.9	94	50.5
25–26.9	29	15.6
27–29.9	24	12.9
≥30	24	12.9
Time since diagnosis of T1DM (years)		
mean ± SD		17.8 ± 12.7
Method of Insulin Administration		
Basal–bolus	139	74.3
Premixed insulins	3	1.6
Continuous subcutaneous insulin infusion (CSII)	45	24.1

**Table 2:** Variables related to T1D decompensations and mortality before and after vaccination.

	Pre-Vaccination		Post-Vaccination		*p*-Value *
	n	%	n	%	
Acute hyperglycemic decompensation	6/161	3.7	7/161	4.3	1
Acute hypoglycemic decompensation	6/161	3.7	6/161	3.7	1
Number of cases of acute hypoglycemic decompensation, mean ± SD	6	2.3 ± 1.4	6	1.3 ± 0.8	
1	1	16.6	5	83.3	
2	4	66.7	0	0.0	
3	0	0.0	1	16.7	
5	1	16.7	0	0.0	
6	0	0.0	0	0.0	
Mortality	1/187	0.53	1/181	0.55	

* Binomial test.

**Table 3:** Acute T1D decompensations depending on the vaccine used.

	Pre-Vaccination		Post-Vaccination		Total	
	n	%	n	%	n	%
Comirnaty						
Acute hyperglycemic decompensation	6/116	5.2	4.3	8/116	6.9	1.0
Acute hypoglycemic decompensation	5/116	4.3	6/116	5.2	8/116	6.9
Spikevax						
Acute hyperglycemic decompensation	0/21	0.0	1/21	4.8	1/21	4.8
Acute hypoglycemic decompensation	0/21	0.0	0/21	0.0	0/21	0.0

**Table 4:** CGM metrics before and after vaccination.

	Pre-Vaccination	Post-Vaccination	*p*-Value *
Average daily scans (n = 85)	9.9 ± 5.6	10.3 ± 6.6	0.314
Mean glucose (n = 113)	158.6 ± 31.7	156.2 ± 32.9	0.200
Coefficient of glycemic variation (CV) (n = 110)	37.3 ± 6.4	36.3 ± 6.8	0.096
Time in glucose range 70–180 mg/dL (TIR) (%) (n = 117)	62.8 ± 16.6	64.4 ± 17.2	0.192
Time with glucose below 70 mg/dL (TBR70) (%) (n = 114)	4.8 ± 4.6	4.3 ± 3.8	0.247
Time with glucose below range 54 mg/dL (TBR54) (%) (n = 112)	1.0 ± 3.3	1.0 ± 3.6	0.900
Number of events of glucose below 70 mg/dL (n = 83)	8.0 ± 7.0	7.6 ± 7.0	0.549
Average duration of hypoglycemic events below 70 mg/dL (min) (n = 83)	66.8 ± 48.6	68.2 ± 58.5	0.799
Time with glucose above 180 mg/dL (TAR180) (%) (n = 107)	21.6 ± 9.9	20.6 ± 9.1	0.188
Time with glucose above 250 mg/dL (TAR250) (%) (n = 107)	10.3 ± 11.5	10.2 ± 11.7	0.970
Glucose management indicator (GMI) (%) (n = 106)	7.2 ± 0.8	7.1 ± 0.8	0.257

Data are expressed in mean ± SD. * *T*-test for paired samples.

## Data Availability

The datasets used and/or analyzed during the current study are available from the corresponding author upon reasonable request.

## Supplementary Materials

The following supporting information can be downloaded at: https://www.mdpi.com/article/10.3390/jcm13071922/s1. Table S1. Baseline sociodemographic and clinical characteristics of the population without follow-up after the first SED1 study phase.

## Abbreviations

ADA, American Diabetes Association; AEMPS, Spanish Agency of Medicines and Medical Devices; BMI, body mass index; CGM, continuous glucose monitoring; CV, cardiovascular; CSII, continuous subcutaneous insulin infusion; DM, diabetes mellitus; EASD, European Association for the Study of Diabetes; EC, Ethics Committee; HbA1c, glycated hemoglobin A1c; IPAQ, International Physical Activity Questionnaire; MET, metabolic equivalent; PA, physical activity; SD, standard deviation; SED, Spanish Diabetes Society; SMBG, self-monitoring of blood glucose; T1D, type 1 diabetes mellitus; T2D, type 2 diabetes mellitus.

## Appendix A

### SED1 Study Investigators

Isabel Serrano Olmedo ^6^; Francisco Tinahones Madueño ^7^; Florentino Carral San Laureano ^8^; Martín López de la Torre ^9^; Alberto Moreno Carazo ^10^; Javier Acha Pérez Hospital ^11^; Orosia Bandrés Nivela ^12^; Edelmiro Menéndez ^2^; Lluís Masmiquel Comas ^13^; Francisca Payeras ^14^; Ignacio Llorente Gómez ^15^; Juan Angel Hernández Bayo ^16^; Coral Montalbán ^17^; Fernando Gómez-Peralta ^1^; Daniel de Luis ^18^; Gonzalo Díaz Soto ^18^; Antonio López-Guzmán ^19^; Estefania Santos Mazo ^20^; Luz Mª López Jiménez ^21^; Visitacion Alvarez ^22^; Benito Blanco Samper ^23^; Ana Chico Ballesteros ^24^; Belen Dalama Gómez ^25^; Ignacio Conget ^4^; Manuel Pérez Maraver ^26^; Berta Soldevila ^27^; Ismael Capel Flores ^28^; Marta Hernández García ^29^; Wifredo Ricart ^30^; Ana Megia Colet ^31^; Elisenda Climent Biescas ^32^; Francisco Javier Ampudia Blasco ^33^; Antonio Hernández Mijares ^34^; Carlos Sánchez Juan ^35^; Antonio Picó ^36^; José Ramón Domínguez Escribano ^37^; Carmiña Fajardo Montañana ^38^; Teresa Pedro ^39^; Pablo Abellán ^40^; Paolo Rossetti ^41^; Francisco M. Morales-Pérez ^42^; Fidel Enciso ^43^; Alfonso Soto González ^44^; Diego Bellido Guerrero ^45^; Reyes Luna Cano ^46^; José Manuel García López ^47^; Víctor M. Andía Melero ^48^; José Alfonso Arranz Martín ^49^; Sharona Azriel Mira ^50^; Marta Botella Serrano ^51^; Miguel Brito Sanfiel ^52^; Alfonso Calle Pascual ^53^; Francisco Javier del Cañizo Gómez ^54^; Manuel Ángel Gargallo Fernández ^54^; Fátima Illán ^55^; Antonio M. Hernández Martínez ^56^; Lluis Forga Llenas ^57^; Sonia Gaztambide ^58^; Clara Rosario Fuentes Gómez ^59^; Amelia Oleaga ^60^; Mª Ángeles Martínez de Salinas Santamaría ^61^; Juan Pedro López-Siguero ^62^; Ana Lucía Gómez-Gila ^63^; Alfonso María Lechuga Sancho ^64^; Marta Ferrer Lozano ^65^; Isolina Riaño Galán ^66^; María Caimari ^67^; Roque Cardona ^68^; María Clemente León ^69^; Gemma Carreras González ^70^; Francisco Javier Arroyo Diez ^71^; Paloma Cabanas Rodríguez ^47^; Belén Roldán ^72^; Noemí González Pérez de Villar ^73^; Purificación Ros Pérez ^52^; Itxaso Rica ^58^; Ignacio Diez López ^59^.

^1^ Endocrinology and Nutrition Unit, Hospital General de Segovia, Segovia, Spain; ^2^ Endocrinology and Nutrition Service; Hospital Universitario Central Asturias, Oviedo, Spain; ^3^ Centro de Salud de Barbastro, Huesca, Spain; ^4^ Endocrinology and Nutrition Unit, Hospital Clínic, Barcelona, Spain; ^5^ SED1 study Investigators, Sociedad Española de Diabetes—SED, Madrid, Spain; ^6^ Hospital Virgen Macarena, Sevilla, Spain; ^7^ Hospital Univ. Virgen De La Victoria, Málaga, Spain; ^8^ Hospital Puerto Real, Cádiz, Spain; ^9^ Hospital Univ. Virgen De Las Nieves, Granada, Spain; ^10^ Hospital de Jaén, Jaén, Spain; ^11^ Hospital Univ. Miguel Servet, Zaragoza, Spain; ^12^ Hospital Royo Vilanova, Zaragoza, Spain; ^13^ Hospital Son Llatzer, Mallorca, Spain; ^14^ Hospital Comarcal de Manacor, Manacor, Spain; ^15^ Hospital Univ. Nuestra Sra Candelaria, Tenerife, Spain; ^16^ Hospital General de La Palma, Breña Alta, Spain; ^17^ Hospital Univ. Marqués De Valdecilla, Santander, Spain; ^18^ Hospital Clínico Univ. Valladolid, Valladolid, Spain; ^19^ Complejo Asistencial De Ávila, Ávila, Spain; ^20^ Hospital Univ. Burgos, Burgos, Spain; ^21^ Complejo Hospitalario Univ. de Albacete, Albacete, Spain; ^22^ Hospital Univ. de Guadalajara, Guadalajara, Spain; ^23^ Hospital Nuestra Señora Del Prado, Talavera, Spain; ^24^ Hospital Sant Pau, Barcelona, Spain; ^25^ Hospital Univ. Vall Hebrón, Barcelona, Spain; ^26^ Hospital Univ. Bellvitge, Barcelona, Spain; ^27^ Hospital Germans Trias I Pujol, Barcelona, Spain; ^28^ Hospital Parc Tauli De Sabadell, Sabadell, Spain; ^29^ Hospital Arnau De Vilanova De Lleida, Lleida, Spain; ^30^ Hospital Josep Trueta, Girona, Spain; ^31^ Hospital JOAN XXIII, Tarragona, Spain; ^32^ Hospital de Mataró, Mataró, Spain; ^33^ Hospital Clinico Univ. De Valencia, Valencia, Spain; ^34^ Hospital Dr. Peset, Valencia, Spain; ^35^ Hospital General Univ. Valencia, Valencia, Spain; ^36^ Hospital General Univ. Alicante, Alicante, Spain; ^37^ Hospital San Juan De Alicante, Alicante, Spain; ^38^ Hospital De La Ribera Alzira, Valencia Spain; ^39^ Hospital Denia, Denia, Spain; ^40^ Complejo Hosp. Univ. Castellon, Castellon, Spain; ^42^ Hospital Gandía, Gandía, Spain; ^42^ Complejo Hospitalario Univ. Badajoz, Badajoz, Spain; ^43^ Complejo Hospitalario Univ. Cáceres, Cáceres, Spain; ^44^ Hospital A Coruña, A Coruña, Spain; ^45^ Hospital de Ferrol, Ferrol, Spain; ^46^ Hospital de Vigo, Vigo, Spain; ^47^ Hospital Clínico de Santiago (Hospital de Conxo), Santiago de Compostela, Spain; ^48^ Hospital Univ. Gregorio Marañon, Madrid, Spain; ^49^ Hospital Univ. La Princesa, Madrid, Spain; ^50^ Hospital Infanta Sofía, San Sebastián de los Reyes, Spain; ^51^ Hospital Univ. Príncipe de Asturias, Alcalá de Henares, Spain; ^52^ Hospital Univ. Puerta de Hierro, Madrid, Spain; ^53^ Hospital Clínico San Carlos, Madrid, Spain; ^54^ Hospital Infanta Leonor, Madrid, Spain; ^55^ Hospital Morales Messeguer, Murcia, Spain; ^56^ Hospital Virgen Arrixaca, Murcia, Spain; ^57^ Complejo Hospitalario Navarra, Pamplona, Spain; ^58^ Hospital de Cruces, Barakaldo, Spain; ^59^ Hospital Univ. Arava, Vitoria, Spain; ^60^ Hospital Basurto, Basurto, Spain; ^61^ Hospital San Pedro, Logroño, Spain; ^62^ Hospital Materno-Infantil - H. Regional Univ. de Málaga, Málaga, Spain; ^63^ Hospital Infantil Virgen del Rocío, Sevilla, Spain; ^64^ Hospital Univ. Puerta del Mar, Cádiz, Spain; ^65^ Hospital Miguel Servet, Zaragoza, Spain; ^66^ Hospital Univ. Central de Asturias, Oviedo, Spain; ^67^ Hospital Son Espases, Palma de Mallorca, Spain; ^68^ Hospital Sant Joan de Deu, Barcelona, Spain; ^69^ Hospital Vall Hebrón, Barcelona, Spain; ^70^ Hospital Sant Pau, Barcelona, Spain; ^71^ Hospital Perpetuo Socorro, Badajoz, Spain; ^72^ Hospital Univ. Ramón y Cajal, Madrid, Spain; ^73^ Hospital Univ. La Paz, Madrid, Spain.
