# Supplementary material for: Metabolic and Clinical Outcomes in Type 1 Diabetes in the COVID-19 Pre- and Post-Vaccination Periods in Spain: The COVID-SED1 Study
Source: J Clin Med. 2024 Mar 26;13(7):1922. doi: 10.3390/jcm13071922 (PMC11012715; doi:10.3390/jcm13071922)
Supplement: Supplementary file 1 [file jcm-13-01922-s001.zip › jcm-2893747-supplementary.pdf]

Supplementary Table S1. Baseline sociodemographic and clinical characteristics of the population without follow-up after the first SED1 study phase.

	n	%
Number of patients	460	
Age (years)		
mean ± SD		36.3 ± 14.0
Age Groups (Years)		
0-13	23	5.0
14-17	21	4.6
18-25	72	15.7
26-49	263	57.2
> 49	81	17.6
Gender, women	250	54.4
Level of education	179	
No studies	1	0.3
Primary education	71	16.3
Secondary education	164	37.8
University studies or similar	163	37.6
Student	35	8.1
Weight (kg)		
mean ± SD		69.2 ± 15.1
Height (cm)		
mean ± SD		166.6 ± 10.5
BMI (kg/m^2)		
mean ± SD		24.8 ± 4.5
BMI grades (kg/m^2)		
< 18,5	17	3.7
18,5-24,9	259	56.7
25-26,9	67	14.7
27-29,9	60	13.1
≥ 30	54	11.8
Time since diagnosis of T1DM (years)		
mean ± SD		17.3 ± 11.7
Method of Insulin Administration		
Basal-bolus	357	77.6
Premixed insulins	5	1.1
Continuous subcutaneous insulin infusion (CSII)	90	19.6
